# Some Remarks on Abdominal Surgery*Read before East Texas Medico-Chirurgical Society, November 29, 1900.

**Published:** 1901-04

**Authors:** A. L. Hathcock

**Affiliations:** Palestine, Texas


For Texas Medical Journal.

### Some Remarks on Abdominal Surgery.*

BY A. L. HATHCOCK, M. D., PALESTINE, TEXAS.

The subject of abdominal surgery assigned me by our program committee is so broad that I have preferred to confine my remarks to some points usually considered of minor importance; and also to a few points about which surgeons differ. In this as well as in every other branch of surgery the careful consideration of all the minor points and close attention to details largely determines the

*Read before East Texas Medico-Chirurgical Society, November 29, 1900.

success of the surgeon. I am not going to offer anything new, nor am I going to trace anew the lines of others' work. I propose to state my own views upon the subject without reference to the views of others who may, or may not, agree with me.

In order to obtain something like a systematic arrangement, I will speak of diagnosis, preparatory treatment, technique and after treatment, again reminding you that I do not propose to give a complete treatise on either, which would require one or more large volumes, instead of a short paper like this.

*Diagnosis.* The ability to make a correct diagnosis is perhaps the most important requisite that the abdominal surgeon should possess. I mean not only the determination of the diseased condition alone, but the proper estimation of all other existing conditions in their bearings upon the case, both general and local. The importance of examination of the various important functions is well known, and yet I have reason to believe that frequently an anesthetic is given without—first, examining the urine; a thing that will cost some one his life if persisted in, for the most trivial operation may be followed by uremia and death in a few days if the patient be already the victim of nephritis at the time he received the anesthetic. Always look into the functions of the kidneys, heart, stomach, liver and lungs, no matter how trivial the operation.

Of equal importance is the exact determination of the local disease, and of its effects upon surrounding structures. Make an earnest effort in each case before going to operation to ascertain just exactly what organ is chiefly affected and what other organs are involved and in what way. Try to determine what amount of adhesions will probably have to be dealt with, and are they old or recent. Do they bind important organs to the tissues that must be removed, and are there probably pus foci? An exact knowledge of these points is so important that no case should be brought to the operating table until they have been made out as accurately as possible, several examinations and anesthesia if necessary should be resorted to in order that the mind may have a clear picture of what will be encountered in the course of the operation. Too frequently, even after our best efforts, we will not find all we expected, but more frequently we will find more than we expected, and we must be prepared for these surprises.

The preparatory treatment will vary with each individual case. Usually several days should be spent in getting the stomach and bowels in the best possible condition by means of light nutritious food, non-irritating laxatives and a glass of hot water taken before each meal. This is particularly important when there exists a mild catarrhal condition of the alimentary canal, sometimes the result of over-eating or of alcoholic drinks.

The skin and kidneys should be flushed by drafts of hot water containing a pinch of salt and a daily bath followed by a brisk rub in all except feeble cases and in these an alcohol bath should constitute a part of the daily routine. If the operation is to be done in a hospital, these several days of preparation should be spent there in order that she may become familiar with her surroundings, get acquainted with her nurse and recover from the nervous erethism incident to her recent arrival in the institution.

The day previous to the operation should be spent in bed and towards its close she should have a laxative dose of castor oil. After the oil has acted a vaginal douche should be given, followed by a bath, clean linen for her person and her bed and finally a dose of potass. bromide to secure a good night's sleep. The next morning an enema is given, the abdomen and vulva are shaved and scrubbed with soap and hot water, douched with alcohol and a 1-1000 solution of corrosive sublimate. The entire field is then protected by sterile towels held in place by a sterile bandage and the patient returned to bed until time for the operation when the abdomen is again thoroughly douched with bichloride solution.

*Technique of Operation.* I make the median incision for operations on the female pelvic organs and the incision for other operations I make the same as the texts advise, provided it suits the individual case. I do not care whether the linea alba is accurately divided or not in making the median incision as I have not been able to see any difference in the results whether the incision follows exactly the white line or falls a little to one or the other side. After opening the peritoneum I think it a good plan to stitch the latter to the skin in order to avoid stripping it off the abdominal walls in the further manipulations. These stitches, if made of heavy silk and left long, serve as retractors for the abdominal walls.

After entering the abdominal cavity the three points that seem to me to be of sufficient importance to mention are:

1st. Work as rapidly as is compatible with neatness and safety, in other words, finish as soon as you can, and

2nd. Avoid injury to the peritoneum by unnecessary handling, rough mopping, hard pulling, and the like.

3rd. Isolate infected areas by packing it off with gauze pads. Make the wound large enough to permit of easy access without undue traction upon its edges, pack back the intestines gently, don't ram them back as if they were a useless organ and that to get them out of the way is the only object. In separating adhesions, do not pull hard on the adherent bowel, bladder, appendix, or any important structure; always let the force of the manipulations fall upon the tissue to be removed. Use the finger or a piece of gauze held in the forceps or sponge holder when possible, cut adhesions only when they are so tough that there is danger of tearing the adherent organs in stripping it loose. Tie thick bands before cutting. If you are eneucleating a fibroid, look out for large veins, especially low down and at the sides of the uterus. In an hysterectomy for fibroid recently I had a very troublesome hemorrhage due to tearing open some large veins at the side of the uterus just when I was ready to pass a ligature around the uterine artery. These enlarged veins will bleed freely from both ends and require a ligature or clamp on the tumor sides as well as on the proximal side.

Remember the course of the ureters—that they lie behind the peritoneum and that there is no danger of including them in a ligature until the lower part of the broad ligature is reached in the region of the internal os, unless the posterior parietal peritoneum is lifted up by a tumor or torn loose in stripping adhesions. Remember, too, that if the posterior parietal peritoneum is lifted up by any means, that the ureters are most likely to come up with it and are apt to be found lying on top of a tumor growing beneath the peritoneum in this situation; or if the posterior peritoneum is torn loose in an effort to free dense adhesions, the ureters are apt to come up with it and are in danger of being torn or ligated. While I have not practiced catheterization of the ureters, I am freely convinced of its great importance in operations involving the tissue about the ureters since a bougie placed in the ureters affords the only means of certainly outlining them in densely filtrated tissues.

After the diseased tissue, whatever it may be, is removed, wipe out the cavity neatly and gently and examine the ligatures to see if they have slipped off or become loosened; never omit this. I have several times tied a ligature as tightly as I could, and after finishing the eneucleation find it so loose that blood was rapidly leaking through the open end of an artery or vein. In such a case catch the vessel with an artery forceps and ligate it separately, after stripping it loose for a short distance. The first ligature may then be cut and removed. I believe it is better always to remove useless ligatures if it is readily done since it leaves just that much less work for nature to do in taking care of these foreign bodies. For the same reason an absorbable ligature is preferable to silk, provided it is as completely sterile and will hold long enough. When the ligatures are all made secure suture all peritoneal rents and tuck the stumps under so as to leave them outside the peritoneal cavity.

The peritoneal toilet will vary according to the amount of bleeding or escape of unclean materials into the cavity. If a very simple operation has been done without any suspicious contamination, the wound will be closed without any further efforts at cleansing. On the other hand, if the peritoneum has been soiled it should be carefully irrigated with warm normal saline solution, clots removed and the solution mopped out several times over. This should be very thoroughly done if pus has escaped, even though it may have been caught on gauze pads.

In regard to drainage, I am not sure that it ever does good. The cases that I have seen die of peritonitis have been those in which drains have been used. To be sure they were bad cases in which the peritoneum had been contaminated and I do not contend that the drains were responsible for the unfavorable result, but I merely wish to say that they did no good in these cases, and I entertain some doubt as to whether they are ever of any service. I do not mean this to apply in cases in which localized abscesses have been opened and treated by the open method, as is sometimes done in appendicitis cases;—here the packing of the cavity with gauze and proper drainage constitutes the more important step in the treatment of such a case.

The closure of the wound in the abdominal wall is a matter concerning which every one has, perhaps, ideas a little different. Many good methods are described and made use of, but I am not sure the more elaborate methods have any advantage over the simple method of passing a single row of sutures through all the layers. The object aimed at is that the sutures be so placed and tied that the different structures are properly coated and held snugly together. In several experiments upon dogs, the single suture for all the layers gave the best results, but it must be remembered that dogs get up and walk about after operations and buried catgut sutures are much more liable to give way than in man and allow the muscles to separate and cause hernia, as was the case in two hernia of my experiments.

My usual method of closing the abdominal wound is by stitching the peritoneum with a continuous catgut suture and bringing the muscles, skin and fascia together with a single silk worm gut suture passed down to the peritoneum. For the purpose of obtaining the views of the surgeons in our section in regard to the use of catgut sutures and ligatures I addressed letters to several surgeons in East Texas and received their replies as follows: Four used silk both within the abdominal cavity and in closing the wound; one used catgut within the abdominal cavity and closed the wound with silk or silk worm gut. One used catgut throughout.

As a dressing, I use anything in the way of sterile gauze—plain sterile gauze is, I believe, as good as any. Of course, the wound must be sterile to start with. I care nothing for dusting powders and never use them in clean wounds. I like a piece of protective to cover the incision to prevent the gauze from sticking, but it must be perforated in order to allow oozing fluids to escape and avoid masceration of the epidermis. Two or three adhesive strips drawn across the dressing keeps it from slipping and a laced many-tailed bandage completes the dressing.

*After Treatment.* The first step in after treatment is taken while the patient is still under the anesthetic and in Trendelenburg's position—one or two quarts of hot saline solution is injected into the rectum to hasten reaction and to aid the action of the skin and kidneys. I consider this the most important remedial agent that we can employ in the after treatment.

I have nothing to say of the general management of the case after operation. My ideas are those generally expressed in the texts on the subject, and there is no point I wish to call attention to, unless it be the state of the pulse as an index of the patient's condition. When tympanitis develops at the end of forty-eight hours and the bowels refuse to act, I know nothing so consoling as a pulse between 70 and 80, and a temperature between 98.6 and 99.5 in spite of the abdominal distress and the stormy outlook as a whole. A placid countenance is also worth quite a little when we are anxiously searching everywhere for some ray of hope.

In closing, gentlemen, I wish again to state that it has not been the intent of this paper to outline a complete abdominal technique, but rather to give my own views upon those points about which surgeons usually differ, and to emphasize a few points that have struck me as not having received the attention they deserve.

